# Advances in understanding glycosyltransferases from a structural perspective

**DOI:** 10.1016/j.sbi.2014.08.012

**Published:** 2014-10

**Authors:** Tracey M Gloster

**Affiliations:** Biomedical Sciences Research Complex, North Haugh, University of St Andrews, St Andrews, Fife KY16 9ST, UK

## Abstract

Glycosyltransferases (GTs), the enzymes that catalyse glycosidic bond formation, create a diverse range of saccharides and glycoconjugates in nature. Understanding GTs at the molecular level, through structural and kinetic studies, is important for gaining insights into their function. In addition, this understanding can help identify those enzymes which are involved in diseases, or that could be engineered to synthesize biologically or medically relevant molecules. This review describes how structural data, obtained in the last 3–4 years, have contributed to our understanding of the mechanisms of action and specificity of GTs. Particular highlights include the structure of a bacterial oligosaccharyltransferase, which provides insights into N-linked glycosylation, the structure of the human *O*-GlcNAc transferase, and the structure of a bacterial integral membrane protein complex that catalyses the synthesis of cellulose, the most abundant organic molecule in the biosphere.

## Highlights

• Glycosyltransferases are the enzymes that catalyse glycosidic bond formation.
• Structural and kinetic studies are important for understanding function.
• Bacterial oligosaccharyltransferase structure aids understanding of N-linked glycosylation.
• Structure of human *O*-GlcNAc transferase gives mechanistic insights.
• Landmark structure of cellulose synthase membrane protein complex.

**Current Opinion in Structural Biology** 2014, **28**:131–141

This review comes from a themed issue on **Carbohydrate-protein interactions and glycosylation**

Edited by **Harry J Gilbert** and **Harry Brumer**

For a complete overview see the Issue and the Editorial

Available online 19th September 2014

**http://dx.doi.org/10.1016/j.sbi.2014.08.012**

0959-440X/© 2014 The Author. Published by Elsevier Ltd. This is an open access article under the CC BY license (http://creativecommons.org/licenses/by/3.0/).

## Introduction

Glycosyltransferases (GTs) are the ubiquitous enzymes responsible for creating the diverse and complex array of oligosaccharides and glycoconjugates found in nature. The chemical diversity and complexity of glycoconjugates, reflecting the various chemical moieties, epimers at each chiral centre, anomeric configuration, linkage position, and branching, requires that the enzymes which catalyze their synthesis, degradation and modification need to be highly specific. GTs catalyse glycosidic bond formation between a sugar and acceptor, which can be a range of biomolecules including other sugars, proteins, lipids and small molecules. The sugar donors are most commonly activated nucleotide sugars, but can also be lipid phosphates and unsubstituted phosphate.

GTs have been classified by sequence homology into 96 families in the Carbohydrate Active enZyme database (CAZy) [1^•^]. The CAZy database provides a highly powerful predictive tool, as the structural fold and mechanism of action are invariant in most of the families. Therefore, where the structure and mechanism of a GT member for a given family has been reported, some assumptions about other members of the family can be made. Substrate specificity, however, is more difficult to predict, and requires experimental characterization of individual GTs. Determining both the sugar donor and acceptor for a GT of unknown function can be challenging, and is one of the reasons there are significantly fewer well characterised GTs than glycoside hydrolases.

GTs catalyse glycosidic bond formation with either overall retention or inversion of anomeric configuration when compared to the stereochemistry in the sugar donor, and the mechanisms employed by these enzymes have been reviewed in detail elsewhere [2^••^,3]. Inverting GTs are generally believed to proceed via a single displacement S_N_2 mechanism with concomitant nucleophilic attack by the acceptor at the anomeric carbon, facilitated by proton transfer to the catalytic base, and leaving group departure [2^••^]. Structural data have shown that several inverting GTs, including the *O*-GlcNAc transferase and oligosaccharyltransferase, which are discussed further below, contain no obvious candidate catalytic base indicating these enzymes use an alternative mechanism. The reaction coordinate employed by retaining GTs has been much debated, and indeed it is possible the mechanism is not conserved for all retaining enzymes. One possibility is a double displacement mechanism via a covalent mechanism, analogous to that used by glycoside hydrolases. A report by Soya *et al*. provided mass spectrometry evidence for the formation of a covalent intermediate between the donor substrate and a cysteine, which had been substituted for the candidate catalytic nucleophile, on two retaining GTs [4]. The more favoured mechanism in the field is an S_N_i (‘internal return’) or S_N_i-like mechanism, which involves interaction between the leaving group and attacking nucleophile on the same face. This mechanism is supported by kinetic isotope effect studies to analyse the structure of the transition state [5^•^] and by computational modelling [6,7].

**Figure 1:**
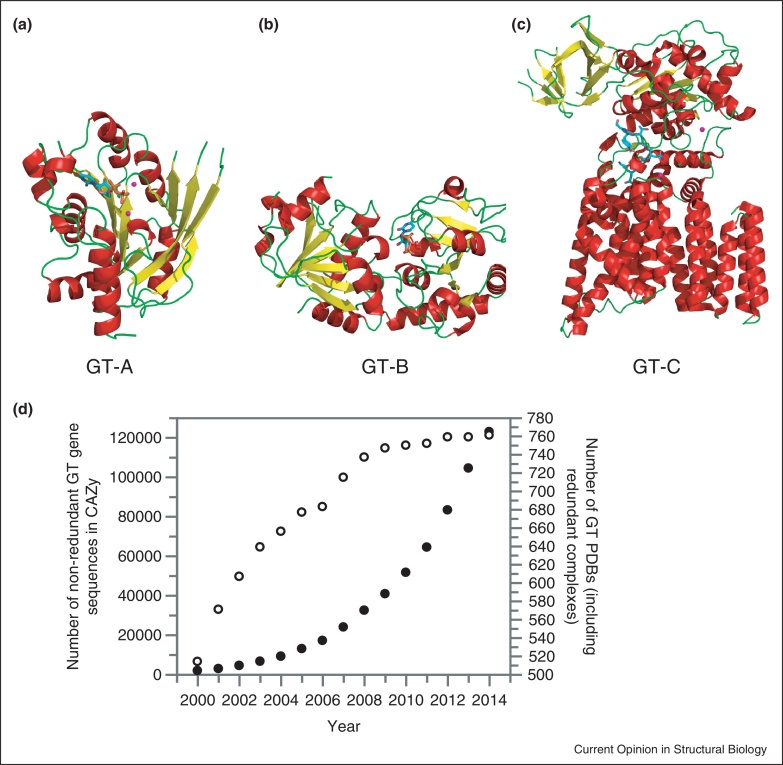
Glycosyltransferases; representative folds and trends in structure solution. Representative structures of (a) GT-A, (b) GT-B, and (c) GT-C glycosyltransferase folds. In each case helices are shown in red, beta-strands in yellow and loops in green. Metal ions, where present, are shown as magenta spheres, and ligands in cyan ball-and-stick representation. The GT-A structure is SpsA from *Bacillus subtilis* in complex with UDP and magnesium (PDB code 1QGS [58]). The GT-B structure is the T4 phage β-glucosyltransferase in complex with UDP (PDB code 2BGU [59]). The GT-C structure is the oligosaccharyltransferase from *Campylobacter lari* in complex with magnesium and peptide substrate (PDB code 3RCE [10^••^]). (d) Graphical representation of the number of non-redundant GT genes curated in the CAZy database (filled circles; left *y* axis) and the number of redundant GT structures (open circles; right *y* axis). Note the number of structures is over-representative of the number of novel GT structures as these figures include ligand complexes, mutants etc. of the same enzyme.

All structures of GTs solved to date adopt one of three folds, termed GT-A, GT-B and GT-C [2^••^] (Figure 1a–c). GT-A enzymes comprise two abutting β/α/β Rossmann-like domains, and are generally divalent metal ion dependent. The metal ion is coordinated by a highly conserved DXD motif within the GT active site and aids leaving group departure by stabilizing the charged phosphate groups in the nucleotide sugar donor. GT-B enzymes consist of two β/α/β Rossmann-like domains that face each other; the active site lies in the cleft between the two domains. GT-B enzymes are generally metal ion independent, with active site residues acting to aid leaving group departure. More recently a third family of enzymes, GT-C, was identified. Structural representatives of these GTs are hydrophobic integral membrane proteins, and perhaps not surprisingly all GT-C enzymes characterised to date use lipid phosphate-linked sugar donors.

Characterisation of GTs lags behind that of the carbohydrate degrading enzymes, glycoside hydrolases. In particular, gaining insights from structure determination is challenging, given the intrinsic difficulty in obtaining GTs in sufficient yield for crystallographic studies and their size precluding analysis by NMR. There are 138 non-redundant GT structures available in the PDB, but these fall into just 38 of the 96 GT CAZy families. Perhaps more worryingly, there has been a significant drop-off in the number of new GT structures (Figure 1d; these figures include redundant structures such as ligand complexes and thus ‘over-represent’ the number of different GT structures) in the last 6–8 years, whilst the number of non-redundant GT sequences has risen exponentially, reflecting the continued increase in genomic and metagenomic sequence information. Nevertheless, there have been several exciting developments in the structural biology of GTs, including structures of three marquee enzymes; the bacterial GT that catalyzes N-linked glycosylation, the human enzyme that mediates O-linked glycosylation of intracellular proteins and an enzyme complex that synthesises cellulose.

## N-linked glycosylation

The most common form of protein glycosylation is the attachment of carbohydrate moieties to asparagine residues (hence N-linked glycosylation). N-linked glycosylation plays a role in various cellular functions including protein folding, quality control, and secretion [8]. The modification is found in all domains of life, although it only exists in a few bacterial species. N-linked glycosylation occurs at the consensus sequence Asn-X-Ser/Thr (where X is any amino acid except proline) on proteins. The substrate donor is a lipid phosphate-linked oligosaccharide; in eukaryotes the lipid carrier is dolichyl pyrophosphate and in bacteria undecaprenyl pyrophosphate. The formation of a glycosidic bond between the amide nitrogen acceptor on the protein and the anomeric carbon on the first sugar molecule is catalysed by the oligosaccharyltransferase (OST), which causes *en bloc* transfer of the oligosaccharide. In eukaryotes OST is a hetero-oligomeric integral membrane protein complex with the catalytic activity residing in the STT3 subunit, whereas the prokaryotic OST (called PglB) comprises a single subunit with homology to STT3 [9].

**Figure 2:**
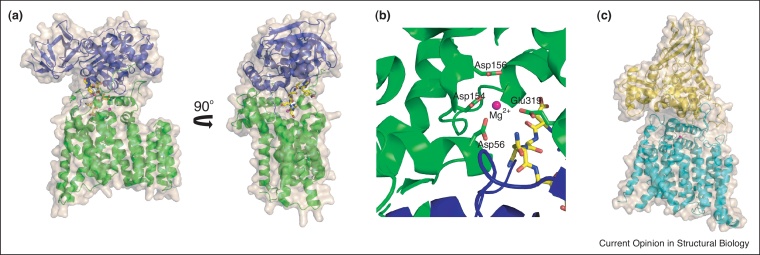
The bacterial oligosaccharyltransferase. (a) Overall structure (in 2 orientations) of the bacterial oligosaccharyltransferase PglB (PDB code 3RCE [10^••^]). The transmembrane domain is shown in green cartoon and the periplasmic domain in blue cartoon, with the surface in beige. The magnesium ion is shown as a magenta sphere and peptide substrate in yellow ball-and-stick representation. (b) Active site of PglB (in the same colouring as (a)) showing the DXD motif coordinating the metal ion, and the catalytically important residues Asp56 and Glu319. (c) Overall structure of the archaeal oligosaccharyltransferase AglB (PDB code 3WAJ [15^•^]). The transmembrane domain is shown in cyan cartoon and the periplasmic domain in yellow cartoon, with the surface in beige. The zinc ion is shown as a magenta sphere.

The structure of PglB from *Campylobacter lari* in complex with an acceptor peptide was solved by Aebi, Locher and colleagues in 2011 [10^••^] (Figure 2a). This landmark structure has added significantly to the mechanistic and functional understanding of N-linked glycosylation in bacteria, and by analogy in eukaryotes. The structure of PglB comprises a helical transmembrane domain and a mixed α/β fold periplasmic domain, which share substantial non-covalent interactions and are both required for binding substrates and catalysis. The peptide substrate binds in a loop with almost a 180° turn, which strongly suggests protein substrates need to possess flexible loops at the protein surface to become N-glycosylated. The serine or threonine residue at the +2 position in the sequon hydrogen bonds with a strictly conserved WWD motif in PglB, and is thought to determine substrate specificity. The active site reveals a metal ion (modelled as Mg^2+^, although later studies show Mn^2+^ is preferred [11]) coordinated to a DXD motif and to Asp56 and Glu319 (Figure 2b); mutagenesis showed these latter residues were highly important for catalysis. There has been much debate about how the amide of the asparagine acceptor acts as a nucleophile given its free electron pair is conjugated, and thus the N—C bond possesses partial double bond character. The structure of PglB shows that Asp56 and Glu319 hydrogen bond with the two amide protons of the asparagine acceptor, which causes rotation of the N—C bond and disrupts the conjugation, thus providing the electron lone pair for nucleophilic attack [10^••^]. This proposed ‘twisted amide’ activation of the acceptor asparagine has gained further support with kinetic measurements and peptide binding experiments with a range of acceptor analogues and with mutated PglB [11].

It is proposed that external loop 5 (EL5), positioned between transmembrane helices 9 and 10, plays an important role in substrate binding and catalysis. Only the C-terminal part of this loop was visible in the structure indicating that EL5 is highly flexible. It is believed that upon binding of the peptide acceptor the C-terminal part of EL5 pins the substrate in place against the periplasmic domain of PglB [10^••^]. Cross-linking experiments demonstrated the necessity for this loop to be able to move in order for both peptide binding and catalysis to occur [12]. The important catalytic residue Glu319 resides in EL5, and so peptide binding and subsequent loop movement would enable the active site to form. Studies into the N-terminal part of EL5 revealed an important motif, called the ‘Tyr-plug’. Mutations of residues in the Tyr-plug, in particular Tyr293, were detrimental to catalysis, but did not affect peptide binding, suggesting the motif played a role in binding the lipid-linked oligosaccharide. It is proposed the Tyr-plug interacts with both the active site residues of PglB and the reducing end sugar of the substrate donor in order to present it in the correct position and conformation for catalysis [12].

More recently the structures of both the C-terminal soluble domain [13,14] and full length OST [15^•^] (Figure 2c) originating from homologues in the archaea *Archaeoglobus fulgidus* (AglB) have been reported, which has allowed parallels to be drawn with the bacterial enzyme. Despite the low sequence identity (<20%) between AglB and PglB, the structures are highly conserved. AglB was crystallised in two different crystal forms, one of which bound sulfate coordinated to the active site metal ion. It is suggested that the sulfate ion mimics the dolichol phosphate binding of the product. Interestingly, the structures confirmed the dynamic role played by EL5 and supported the PglB studies. In the structure with sulfate bound the loop was completely disordered, whereas it was ordered in the other (lower resolution) crystal form in the absence of any bound ligands. The authors propose a model where EL5 is structured in the absence of either substrate, but is conformationally flexible upon binding of substrates. One of the AglB crystal forms bound Zn^2+^, consistent with the observation that the enzyme is more active with Zn^2+^ than Mg^2+^ or Mn^2+^ [15^•^]. This is in contrast to the metal preference displayed by PglB [11]. Very recently, the first structure of one of the eukaryotic OST subunits, N33/Tusc3, was reported. The subunit has a membrane-anchored N-terminal thioredoxin domain, which may form mixed disulphide bonds with OST substrates, and thus increase the efficiency of glycosylation [16].

A different form of N-linked glycosylation is present in some bacteria, including *Haemophilus influenzae*, which involves addition of a single glucose or galactose to asparagine residues on specific adhesion proteins; the glucose can be further elaborated to diglucose [17]. A soluble GT-41 enzyme (same family as the *O*-GlcNAc transferase, see below) is responsible for glycosyltransfer of both the single sugar modification and the chain extension. The structure of the enzyme from *Actinobacillus pleuropneumoniae* has been reported, and revealed an N-terminal all α-domain (in place of the tetratricopeptide repeat domain in OGT), which likely binds the acceptor protein, and a C-terminal GT-B domain that contains the active site [18].

## *O*-GlcNAc transferase

An *O*-linked N-acetylglucosamine residue (*O*-GlcNAc) is added to serine or threonine residues (via a β-linkage) in a variety of nucleocytoplasmic proteins in metazoans. Whilst the function of the *O*-GlcNAc modification is not fully understood, it has been proposed to play roles in gene regulation, signalling, and nutrient sensing, and is implicated in a number of diseases [19]. Unlike N-linked glycosylation, the *O*-GlcNAc modification does not occur at a defined consensus sequence in the protein substrate. The addition of *O*-GlcNAc to proteins is performed by the *O*-GlcNAc transferase (OGT), using uridine diphosphate *N*-acetyl glucosamine (UDP-GlcNAc) as the substrate donor, and proceeds with inversion of anomeric configuration. OGT comprises an N-terminal tetratricopeptide repeat (TPR) domain, which is important for interactions with protein substrates and likely aids selectivity, and a C-terminal catalytic domain. Whilst the structures of the TPR domain from human OGT [20] and bacterial homologues of OGT [21,22] had been reported, the molecular details of the human catalytic domain remained elusive.

**Figure 3:**
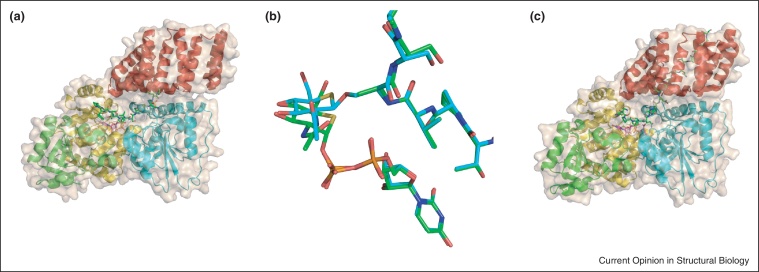
The human *O*-GlcNAc transferase. (a) Overall structure of the human *O*-GlcNAc transferase (PDB code 3PE4 [23^••^]). The truncated tetratricopeptide repeat domain (4.5 of the 11.5 repeats were present in the structure) is shown in red cartoon, the N-terminal catalytic domain in cyan cartoon, the intervening domain in green cartoon and the C-terminal catalytic domain in yellow cartoon, with the surface in beige. UDP is shown in magenta ball-and-stick representation and the peptide substrate in green. (b) Overlap of OGT ternary complex (with UDP-5SGlcNAc and peptide acceptor; green; PDB code 4GYY [26^•^]) and product complex (with UDP and 5SGlcNAc-glycopeptide; cyan; PDB code 4GZ3 [26^•^]). (c) Structure of OGT (with colouring as in (a)) in complex with UDP-5SGlcNAc in magenta ball-and-stick representation and residues 1-26 of HCF-1 peptide in green (PDB code 4N3B [31^••^]).

The milestone structure of human OGT was solved in 2011 by Walker and colleagues, comprising the catalytic domain and 4.5 (of 11.5) TPR units [23^••^]. As predicted for a GT-B enzyme, OGT has two Rossmann-like domains, although the N-terminal domain has two additional helices that contribute to the active site. Between the two Rossmann-like domains is the ‘intervening domain’, which displays a novel fold with a 7-stranded β-sheet core and flanking α-helices and offers a large basic surface. A transitional helix links the TPR and catalytic domains; the TPR domain lies on the upper surface of the catalytic region (Figure 3a). The structure of OGT was solved in complex with UDP and with UDP and a substrate peptide, which revealed important features of the active site. In the presence of peptide the cleft between the TPR and catalytic domains is wider to accommodate the peptide, and suggests a ‘hinge’ at the interface between the two domains exposes the active site. The peptide interacts with OGT predominantly through contacts with the backbone, which allows variability in sequence, but does suggest the protein substrate needs to be presented in a flexible region or loop. The peptide substrate binds over the nucleotide binding pocket, suggesting the substrate donor UDP-GlcNAc has to bind first, and indeed the α-phosphate helps to orientate the peptide [23^••^].

The identity of the catalytic base in OGT has remained controversial. The structures of the bacterial homologues suggested His558 [21] or a tyrosine [22] as possible candidates, while the human OGT structure suggested His498 was more likely [23^••^]. Small molecule inhibitors and mechanistic probes have given some insights. A dicarbamate-containing inhibitor, identified in a high-throughput screen, irreversibly inactivated OGT, and the structure revealed the inhibitor cross-linked two active site residues [24]. The authors proposed the neutral cross-link mimicked the position of the diphosphate group of the substrate donor. 5-thio derived UDP-GlcNAc (UDP-5SGlcNAc) is a substrate donor mimic that binds to OGT and is turned over significantly more slowly than UDP-GlcNAc [25]. The ternary structure of OGT in complex with UDP-5SGlcNAc and acceptor peptide (together with either a mutant enzyme or pseudo-substrate peptide to allow capture of the complex) was solved by two groups [26^•^,27^•^], and provided insights into OGT catalysis. Superposition of the ternary and product complexes was consistent with an electrophilic migration mechanism where an oxocarbenium ion-like species moves from the leaving group to the acceptor [26^•^,27^•^] (Figure 3b). The structures did, however, discount all of the previously proposed nucleophile candidates as being in unsuitable positions, and offered no further residues in the OGT active site that might fulfil this role. The groups proposed different scenarios for how OGT may overcome deprotonation of the serine in the acceptor peptide. Lazarus *et al*. suggest this role may be performed by a chain of water molecules linking the proton to a conserved aspartate residue [26^•^], similar to the Grotthus mechanism proposed for some inverting glycoside hydrolases [28], whilst Schimpl *et al*. postulate the α-phosphate of the nucleotide sugar donor could fulfil the same function [27^•^]. The structure of the OGT ternary complex has, therefore, perhaps raised more questions than it has answered [29], but still has furthered our mechanistic knowledge of OGT.

More recently, structural and mechanistic insights into the role played by OGT in cell cycle progression have been reported. OGT appears capable of proteolytically cleaving repeat units of host cell factor-1 (HCF-1), a transcriptional regulator of the cell cycle, in the presence of UDP-GlcNAc [30]. Cleavage occurs at a CET motif, between the cysteine and glutamate, which is converted to pyroglutamate [31^••^]. The structure of OGT in complex with a HCF-1 repeat showed a threonine rich region of the repeat bound, in an extended manner, to the TPR domain of OGT, in particular via a series of asparagine residues. In the absence of UDP-GlcNAc, only the C-terminal region of the HCF-1 repeat could be visualised, but the addition of UDP-5SGlcNAc stabilised the complex to allow the interactions with the N-terminus to be observed (Figure 3c). The N-terminal region of the repeat makes extensive interactions with UDP-5SGlcNAc, and, perhaps surprisingly, the region of the peptide that is cleaved binds in the same manner as a peptide that is glycosylated. In fact the glutamate of the CET motif aligns perfectly with the serine that is glycosylated, and substitution of glutamate for serine in this motif switched OGT activity on the protein from proteolysis to glycosylation. The authors suggest that OGT may actually glycosylate the glutamate prior to cleavage, but further investigations are required to understand the mechanism [31^••^].

## Cellulose biosynthesis

The molecular understanding of how cellulose is synthesized has remained elusive, despite it being the most abundant polymer on earth. The formation of cellulose, a linear polysaccharide comprising β-linked 1,4 glucose, is performed by cellulose synthase using the substrate donor UDP-glucose (UDP-Glc). Plants, algae and some bacteria possess several cellulose synthases. Whilst cellulose is most commonly associated with plant cell walls, some bacteria also produce the polysaccharide to aid biofilm formation. In bacteria, cellulose biosynthesis and transport is mediated by a protein complex comprising three subunits: BcsA, BcsB, and BcsC. BcsA is an integral inner membrane protein and possesses the GT activity, BcsB is a membrane-associated periplasmic protein, and BcsC comprises an 18-strand β-barrel and is located in the outer membrane.

**Figure 4:**
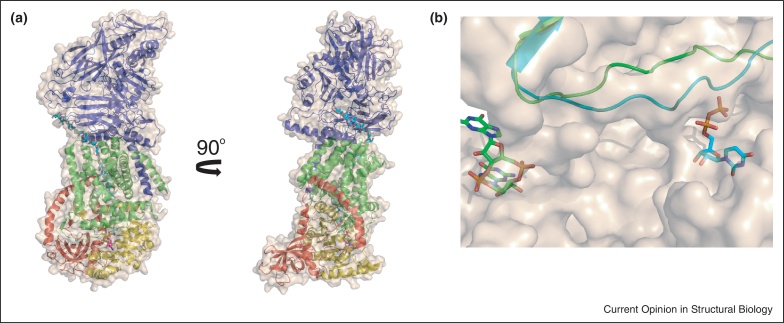
Bacterial cellulose synthase. (a) Overall structure (in 2 orientations) of the bacterial cellulose synthase complex (PDB code 4HG6 [32^••^]). The transmembrane domain of BcsA is shown in green cartoon, the GT domain in yellow cartoon, and the C-terminal domain in red cartoon; BcsB is shown in blue. The overall surface is shown in beige. UDP is shown in magenta ball-and-stick representation and the cellulose fragment in cyan. (b) Overlap of cellulose synthase in the presence of UDP (cyan cartoon/ball-and-stick; PDB code 4HG6 [32^••^]) and presence of cyclic-di-GMP (green cartoon/ball-and-stick; PDB code 4P02 [35]).

The ground-breaking structure of BcsA and BcsB from *Rhodobacter sphaeroides* has provided insights into the mechanism and translocation of cellulose [32^••^], and has implications for the plant equivalents [33]. BcsA and BcsB form a stoichiometric complex, with a large interface (Figure 4a). BcsA possesses 8 transmembrane helices with a large intracellular loop between helices 4 and 5 that forms the GT domain (belonging to family GT-2). BcsB is a predominantly β-stranded periplasmic protein, with a single transmembrane anchor that interacts with BcsA. Strikingly, the structure was solved in complex with a cellulose fragment of 18 glucose residues, which was presumably being synthesized before or during crystallization. There was also weak density corresponding to a UDP molecule, suggesting the structure captured the reaction coordinate following glycosyl transfer [32^••^].

The GT domain of BcsA adopts a GT-A fold comprising seven β-strands surrounded by seven α-helices, and contains signatures characteristic of processive GTs. The structure reveals that two conserved aspartate residues coordinate UDP, and a third aspartate (part of an invariant TED motif) likely functions as the catalytic base. A Q(Q/R)XRW motif binds the terminal disaccharide acceptor of the glucan chain, and is located at the cytoplasmic entry to the channel. The narrow channel for translocation of cellulose is formed by transmembrane helices 3–8 and appears to accommodate 10 glucose residues. On exiting from the channel, the cellulose fragment kinks sideways. The size of the substrate binding pocket indicates that one glucose residue is added to the polysaccharide at a time, and indeed the narrow dimensions of the channel support the proposal that it would also only be possible to translocate the chain one residue at a time [32^••^].

BsbB has four domains, two jellyroll and two flavodoxin-like folds; the jellyroll domains show high similarity to carbohydrate binding modules (CBMs) that are often found in a tandem modular arrangement with carbohydrate degrading enzymes [34]. The domains in BscB, termed CBD1 and CBD2, are likely to bind to the oligosaccharide during translocation [32^••^].

BcsA activity is stimulated by cyclic-di-guanine monophosphate (GMP), which is mediated by a PilZ domain located at the C-terminus of BcsA, next to the GT domain. It has been predicted that binding of cyclic-di-GMP causes a conformational change to allow UDP-Glc access to the catalytic site [32^••^], but very recent structural studies have provided insights into the mechanism [35]. The structure of the BcsA–BcsB complex, with a cellulose fragment bound, was solved in complex with cyclic-di-GMP both in the absence and presence of UDP. Cyclic-di-GMP interacted with a number of conserved residues in the PilZ domain of BcsA. Binding of cyclic-di-GMP caused conformational changes in the PilZ domain, in particular disruption of an Arg-Glu salt bridge, resulting in the movement of a ‘gating loop’ that blocked the entrance to the GT domain which caused it to adopt a different conformation (Figure 4b). This ‘open’ structure ensured there was sufficient space for UDP-Glc to bind. In the presence of UDP (and cyclic-di-GMP), this loop moved 15 Å compared to the ‘open’ conformation and was inserted into the active site where it is thought to play a role in positioning the substrate donor [35].

## Protein fucosylation

Protein fucosylation is a post-translational modification involving addition of an *O*-linked fucose (which can be further elaborated by other sugars) to serine or threonine residues in proteins with cysteine rich motifs such as Notch and thrombospondin 1. The transfer of fucose is catalysed by the endoplasmic reticulum localised GTs protein *O*-fucosyltransferase 1 (POFUT1) and protein *O*-fucosyltransferase 2 (POFUT2), using the substrate donor guanine diphosphate fucose (GDP-Fuc). The protein acceptor for POFUT1 is epidermal growth factor (EGF)-like repeats, and for POFUT2 is thrombospondin type 1 repeats (TSR) [36]. Structural studies into both fucosyltransferases have been reported recently.

POFUT1 is a GT-65 inverting enzyme that is not metal dependent [37]. The structure of POFUT1 from *Caenorhabditis elegans* revealed a GT-B fold, and a substrate-bound complex with GDP-Fuc showed it was located in a cavity at the interface between the two Rossmann-like folds. GDP-Fuc points out to a solvent exposed positively charged pocket, which docking studies show would bind the negatively charged EGF repeats from Notch. Mutagenesis studies revealed that an active site arginine played a key role in binding the substrate donor and in catalysis, and an asparagine residue was important for binding to fucose and possibly positioning the nucleophilic water during catalysis. The active site offers no candidate catalytic base, and the authors propose the β-phosphate of GDP-Fuc may fulfil this role [37].

Human POFUT2 is a GT-68 inverting GT which is structurally similar to POFUT1 [38]. The structure, in concert with biochemical studies, indicates that specificity for TSRs is conferred by a few conserved residues that form key three-dimensional structural elements. Studies have indicated POFUT2 activity is enhanced in the presence of metal ions, although no metal was observed in the structure. The structure and mutagenesis studies suggest a glutamate may act as the catalytic base [38], possibly in a different mechanism for that proposed for POFUT1. Further investigations are needed to build upon these studies in order to probe the substrate specificity and catalytic mechanism of these related fucosyltransferases with unique specificities.

## Natural product biosynthesis

There is significant interest in the pharmacological and biomedical potential of GTs, through their capacity to synthesise natural products containing sugar residues. Engineering of GTs to synthesize molecules with different chemistries is appealing, but requires an understanding of how structure is related to function. A comprehensive study reported the structures of the four GTs involved in the calicheamicin biosynthetic pathway, which provides rare insights into the complete biosynthesis of a molecule [39]. The four GTs show high structural homology, despite low sequence identity, but subtle differences in acceptor molecule recognition demonstrate how they have evolved their specific chemistries and regioselectivity, and indicates regions where bioengineering could evolve these activities further [39].

Structures of other GTs involved in the biosynthesis of natural products have been solved and provided insights into their mechanism. The GT SnogD from *Streptomyces nogalater* is responsible for adding a nogalamine moiety in the final steps of the biosynthesis of the aromatic polyketide nogalamycin. Nogalamine is attached to the polyketide via both an *O*-glycosidic bond and C—C linkage. The structure of SnogD, in complex with nucleotide, supports the mechanism for the formation of the *O*-glycosidic bond, but it is less clear how SnogD catalyses the synthesis of a C—C bond, which may in fact be generated by a different enzyme [40]. The GTs capable of transferring glucose to anthocyanidins [41] and of transferring olivose to a tetracycline-like scaffold via the formation of a C—C bond [42] have also been investigated. Advances have been made in understanding the biosynthesis of the important antibiotic erythromycin D. The GT EryCIII transfers desosamine onto the macrolide scaffold, but studies showed the presence of an activator protein, EryCII, was crucial for GT activity. The structure of the complex revealed an unusual heterotetrameric elongated quaternary structure [43].

## Glycogenin and glycogen synthase

Glycogenin is a metal dependent GT-8 retaining enzyme responsible for synthesizing 6-10 α-1,4-linked glucose residues using UDP-Glc as the substrate donor. Glycogenin employs an unusual mechanism as the oligosaccharide chain is synthesized while attached to the enzyme, via a tyrosine residue. The tyrosine acts as the acceptor in the first reaction, and the Tyr-glucoside in subsequent reactions. Once the short fragment is synthesized, it acts as the primer for glycogen synthesis, performed by glycogen synthase and the glycogen branching enzyme. Glycogenin displays an N-terminal single Rossmann-like fold and operates as an obligate dimer. Crystallographic snapshots of glycogenin at different stages of its catalytic cycle were captured, and demonstrated large conformational changes involving a 30 residue ‘lid’ that covers the active site, induced by UDP-Glc binding [44^•^]. This lid is important for guiding the oligosaccharide chain acceptor (either inter-molecularly or intra-molecularly) into the active site in the correct position for catalysis. Very recently insights have been gained into the interactions between glycogenin and glycogen synthase (a GT-3 enzyme) from *C. elegans*. The C-terminal ‘tail’ of glycogenin is of undefined structure, but the structure of this peptide in complex with glycogen synthase was solved. The interactions defined a new position for allosteric regulation of glycogen synthase, and experiments showed the presence of the fragment is critical for glycogen synthesis [45].

## Other highlights

The structure of human β-galactoside α-2,6-sialyltransferase, which caps the terminal galactose residues in N-glycans with sialic acid, was reported in complex with its product, cytidine monophosphate [46]. Fortuitously a glycan on the protein of a symmetry related molecule was bound in the active site, which revealed likely interactions between the enzyme and acceptor. The structure of the rat α-2,6-sialyltransferase has also been reported recently [47]. Kinetic and structural insights have been offered on the GT-42 bifunctional sialyltransferase (capable of transferring sialic acid α2,3-linked to galactose of lipooligosaccharide and a further sialic acid α2,8-linked to the first product) from *Campylobacter jejuni* [48] and the GT-52 α2,3/α2,6 lipooligosaccharide sialyltransferase from *Neisseria meningitidis* [49]. In the latter structure a novel domain swap was observed to form a functional homodimer.

A significant advance in elucidating the biosynthesis of lipopolysaccharide was made with the structure of Waa (a family GT-30 enzyme) from *Aquifex aeolicus*, which catalyses the transfer of 3-deoxy-d-manno-oct-2-ulosonic acid to the lipid A precursor of lipooligosaccharide. The structure revealed an N-terminal domain with a hydrophobic surface-exposed patch, which is proposed to embed Waa into the membrane and enable lipid A binding. Large conformational changes induced by substrate binding would then bring the substrate donor and acceptor into position for catalysis [50].

Family GT-6 contains GTs that catalyse formation of α-1,3 linkages between galactose or N-acetylgalactosamine (GalNAc) and β-linked galactose or GalNAc acceptor substrates. Mammalian members of family GT-6 are metal dependent, and contain the DXD motif, whereas a number of the bacterial species assigned to the same family have a conserved NXN motif and are metal independent. The structural basis for this has been described with two family GT-6 structures from *Bacteroides ovatus* [51,52]. There is a high degree of structural homology between the bacterial and mammalian GT-6 enzymes, with equivalent residues for binding substrates.

An insightful study by Tsutsai and colleagues reported the structures of both the human and *Drosophila* metal dependent β-1,4-galactosyltransferase responsible for catalysing transfer of galactose from UDP-galactose (UDP-Gal) to xylose in the core tetrasaccharide of a proteoglycan acceptor [53]. The structures of various complexes with product (UDP), substrate donor (UDP-Gal) and a ternary complex with substrate donor and acceptor (with an inactive mutant enzyme) demonstrated the conformational changes during catalysis. The substrate(s) bound complexes underwent rearrangement of two loops to move from an ‘open’ to ‘closed’ state, and involved reorganisation of important residues in the catalytic pocket. The ternary complex is consistent with an S_N2_ mechanism being employed for catalysis [53].

Striebeck *et al*. reported the structure of a fungal mannosyltransferase, the first from family GT-62. The enzyme adopts a GT-A fold, with an unusual hairpin loop extension. Interestingly, the presence of the GT and its product is necessary for the activity of a second mannosyltransferase and therefore may also play a secondary ‘priming’ role [54]. The structure of GlgE, a (1 → 4)-α-d-glucan:phosphate α-d-maltosyltransferase from *Streptomyces coelicolor*, was reported; the enzyme has the same properties as the *Mycobacterium tuberculosis* GlgE which is a validated drug target for the treatment of tuberculosis [55]. The structure and kinetic studies were described for a bifunctional polymerizing galactofuranosyltransferase that synthesizes galactan in the mycobacterial cell wall, which demonstrated how the enzyme could generate alternate β(1 → 5) and β(1 → 6) linkages within the same active site [56]. In addition, the structure of a glucosyltransferase responsible for modification of *Streptococcal* and *Staphylococcal* serine-rich repeat glycoproteins revealed a novel GT family [57], which as yet has not been assigned in the CAZy database.

## Concluding remarks

GTs are, without question, difficult to study. GTs are difficult to produce in high yield in recombinant form, particularly those that are membrane associated or are integral membrane proteins, and can be difficult to crystallise as they are often multi-domain proteins and capable of undergoing substantial conformational changes. In addition, GTs are often difficult to characterise as these bimolecular enzymes require the identification of both substrate donor and acceptor. The significant drop-off in the number of new GT structures solved compared to the exponential growth in non-redundant gene sequences is a cause for concern (Figure 1d). Despite the challenges, however, significant advances have been made in understanding a plethora of GTs in the last few years using structural techniques. The three-dimensional structural information has guided kinetic and mutagenesis studies leading to significant advances in understanding of catalytic mechanism and substrate binding. In turn, this will enable further studies such as development of inhibitors against targets that are medically relevant or bioengineering of those enzymes that are capable of synthesizing novel and industrially or medically significant biomolecules. The significant steps forward on several key GTs, for example those involved in N-linked glycosylation, the *O*-GlcNAc modification, and cellulose biosynthesis, represents a substantial advance for the field. These structural breakthroughs underpin our understanding of the molecular details of these enzymes, which will now impact on medical or biotechnological processes. The carbohydrate enzyme structural community should take heed that elucidating GT structures previously considered as the pinnacle of difficulty is possible, and is vital to furthering our understanding of this key class of enzyme.

## References and recommended reading

Papers of particular interest, published within the period of review, have been highlighted as:

• of special interest
•• of outstanding interest
